# Effect of patient-specific instruments compared with conventional instruments in total knee arthroplasty: a randomized controlled trial

**DOI:** 10.2340/17453674.2025.44924

**Published:** 2025-12-02

**Authors:** Jakob HERMODSSON, Tuuli SAARI, Bita SHAREGHI, Maziar MOHADDES, Anna NILSDOTTER, Johan KÄRRHOLM

**Affiliations:** 1 Department of Orthopedics, Sahlgrenska University Hospital, Mölndal; 2 Department of Orthopaedics, Institute of Clinical Sciences, Sahlgrenska Academy, Gothenburg; 3 Department of Orthopaedics, Skåne University Hospital, Hässleholm;; 4 Unit for Orthopaedics, Department of Clinical Sciences Lund, Lund University, Lund, Sweden

## Abstract

**Background and purpose:**

Patient-specific instrumentation (PSI) in total knee arthroplasty (TKA) aims to improve implant alignment and clinical outcomes, but its effectiveness remains uncertain. We aimed to compare whether PSI was superior to conventional instrumentation (CVI) in TKA. The primary outcome was the Oxford Knee Score (OKS) at 2 years with assessments of additional clinical outcomes up to 5 years after surgery.

**Methods:**

This study included 70 knees with primary osteoarthritis randomized (1:1) to undergo TKA using either PSI or CVI. Outcomes were evaluated using patient-reported outcome measures (PROMs), radiographic alignment, and radiostereometric analysis (RSA) of migration over the full follow-up period of 5 years.

**Results:**

68 knees underwent surgery as per protocol. At 2 years, 29 knees in the PSI group and 36 in the CVI group were assessed with the OKS, which improved significantly by 21.2 in the PSI group and 18.2 in the CVI group (mean difference [MD] 2.1; 95% confidence interval [CI] –1.5 to 5.7). PSI resulted in slightly increased tibial varus alignment (MD –1.4°; CI –2.3° to –0.4°), migrated slightly more into varus (mean difference at 2 years: –0.28 mm, CI –0.54 to –0.03 mm), and showed higher maximum total point motion (MTPM) during the period 1 to 2 years (mean difference: 0.13 mm; CI 0.01–0.25). At 5 years, 3 knees had been revised (PSI: 2; CVI: 1).

**Conclusion:**

PSI did not demonstrate superior outcomes compared with CVI. These findings suggest that PSI may not provide significant benefits over conventional techniques in routine TKA.

Despite excellent implant survivorship, dissatisfaction is reported after total knee arthroplasty (TKA) in up to 20% of cases. Inadequate patient education and expectation management, residual pain, and functional limitations sometimes related to implant positioning are suggested reasons [1]. Malalignment may increase strain and overload components, soft tissues, or native bone stock, leading to pain, improper function, and implant failure [2].

TKA traditionally relies on jig-based instrumentation, which may not always accommodate individual anatomical variation. Patient-specific instrumentation (PSI) was developed to enhance alignment by tailoring surgical guides to each patient’s unique anatomy. The manufacturing of PSI guides is based on different preoperative imaging modalities (e.g., CT or MRI) and diverse production technologies, making the systems somewhat heterogeneous. PSI may also be useful in cases with altered femoral or tibial anatomy, such as prior trauma, extreme curvature, or preexisting implants.

Earlier trials have yielded conflicting results, likely due to variability in PSI designs and definitions of favorable positioning. Although some studies report improved radiological outcomes, these findings rarely correlate with better clinical outcomes [3-12].

We aimed to compare whether PSI was superior to conventional jig-based instrumentation (CVI) in routine TKA for osteoarthritis (OA), with a primary objective to compare the difference in Oxford Knee Score (OKS) at 2 years from surgery and secondary assessments of other clinical outcomes, radiological implant positioning, and tibial component migration up to 5 years from surgery.

## Methods

### Study design

The study was designed as a randomized controlled trial (RCT). Randomization and sequence allocation were performed by a study nurse using sealed envelopes opened by the study nurse after obtaining written informed consent. Stratification was performed based on presence of preoperative varus or valgus alignment with an expected distribution of 4 to 1. For patients undergoing bilateral TKA, the first knee was randomized, and the second was operated on using the opposite instrumentation method. Neither surgeons nor patients were blinded, the latter due to extra costs of a clinically unnecessary preoperative MRI in the control group.

The study is reported according to CONSORT guidelines, and was registered at ClinicalTrials.gov (NCT06720012).

### Participants

70 knees diagnosed with primary OA were recruited from the Department of Orthopedics at Sahlgrenska University Hospital, Mölndal, Sweden. Patients were randomized (1:1) to undergo TKA using either CVI or PSI based on preoperative MRI scans.

Patients with primary OA of the knee who had depleted conservative management strategies and were on our waiting list for a TKA between 2014 and 2017 were screened for the study. Inclusion criteria were medial or lateral primary OA Ahlbäck Grade 2–4, varus or valgus deformity ≤ 10°, extension defect ≤ 10°, age 40–75 years, body mass index (BMI) < 35, American Society of Anesthesiologists Physical Status (ASA) score I–III, coming from independent living in own home and able and willing to undergo MRI scan, full-leg standing radiographs, and follow-up with RSA. Exclusion criteria were cortisone treatment during the last 6 months before operation, neurological diseases with symptoms, stroke with sequelae, endocrine diseases with symptoms, OA of the hip with symptoms, ongoing infection, any metal within 150 mm from the joint line on the side to be operated on, or unwillingness or inability to participate in the follow-up. Patients were recruited by orthopedic surgeons or a study nurse.

### Surgical procedure

All surgeries were performed or supervised by experienced knee arthroplasty surgeons following standard protocols and without the use of a tourniquet. A cemented TKA (NexGen Complete Knee Solution, Zimmer Biomet, Warsaw, IN, USA) was used in all cases, with either conventional or patient-specific instrumentation. The choice of a patellar component, and cruciate-retaining (CR) or posterior-stabilized (LPS) prosthesis was at the surgeon’s discretion. For those randomized to the PSI group, custom guides were created based on a preoperative MRI protocol (Zimmer Patient Specific Instrumentation for NexGen Complete Knee Solution, Zimmer Biomet, Warsaw, IN, USA in collaboration with Materialise NV, Leuven, Belgium) to optimize the placement of the components without the need for intramedullary alignment. Local anesthetic was infiltrated periarticularly during the procedure. Patients were mobilized within 1 day of surgery and discharged according to hospital routine. During surgery and outside of standard surgical protocol, 5–10 tantalum markers 0.8 and 1.0 mm in size were placed in the proximal tibia and polyethylene insert to allow for radiostereometric analysis (RSA) of implant migration.

### Clinical outcomes

The primary outcome was patient-reported pain, knee function, and stability assessed using the Oxford Knee Score (OKS) at 2 years, an instrument validated for use in Sweden for measuring functional outcomes following knee arthroplasty [13]. A minimal important change (MIC) value of 8, as calculated by Ingelsrud et al. [14], and representing significant clinical improvement on an individual level, was used to assess the number of significantly improved patients in each group. For the minimally clinically important difference (MCID) a value of 5 was used as calculated by Clement et al. [15].

Secondary clinical outcomes were quality of life assessed using the EQ-5D-3L (measures health-related quality of life across 5 domains; index range –0.59 to 1.00, where 1.00 indicates full health, using Swedish value tariffs) [16], EQ VAS (measures self-rated overall health on a visual analog scale from 0 to 100, with 100 representing the best imaginable health), and University of California, Los Angeles (UCLA) Activity Scale (measures physical activity level, scored from 1 = wholly inactive to 10 = regular participation in impact sports; higher scores indicate greater activity) [17]. Length of stay, perioperative blood loss, and surgical time were recorded at the time of surgery. Follow-up data up to 5 years are presented. The study protocol originally included the Knee Society Score (KSS) but it was excluded from the analysis due to an error in the patient-completed forms, which affected the scoring accuracy and compromised the integrity of the instrument. Pain and satisfaction VAS scores were planned according to the study protocol but were also excluded from this analysis.

### Radiographic and CT evaluations

Radiographic evaluation as additional secondary outcome included conventional imaging (anteroposterior [AP], lateral views, and full-leg standing radiographs) and CT scans. Plain radiographs were used to assess postoperative coronal alignment (hip–knee–ankle [HKA] angle) and the positioning of TKA components in coronal and sagittal planes. Axial, or rotational, alignment of components was assessed postoperatively using CT. We used the anatomical trans-epicondylar axis for determining the femoral component rotation, the posterior condyles of the tibia, and a lateral landmark for determining the tibial component rotation [18]. Implant alignment and radiolucent lines were measured according to the Modern Knee Society Radiographic Evaluation System [19]. Radiolucent lines, defined as areas of decreased density between the bone and cement or bone and implant interface, were evaluated on AP and lateral radiographs at baseline, 2 years, and 5 years. 2 independent assessors made repeated measurements using Sectra Workstation IDS7 (Sectra AB, Linköping, Sweden) plain X-rays, AGFA XERO Viewer (AGFA HealthCare N.V., Mortsel, Belgium) for CT scans, and Mdesk (RSA Biomedical, Umeå, Sweden) for plain radiographs.

The radiologic measurements showed good inter-observer agreement with an ICC of 0.88 (95% confidence interval [CI] 0.71–0.83) for radiolucent lines and 0.88 (CI 0.79–0.93) and 0.84 (CI 0.72– 0.91) for the femoral and tibial component rotations, respectively.

### Radiostereometric analysis (RSA)

**Figure 1:**
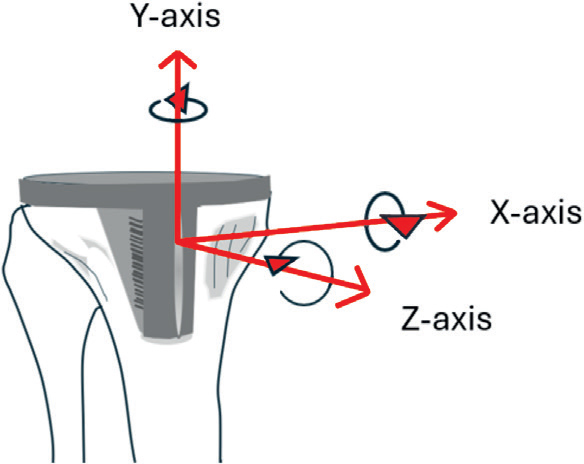
Illustration of tibial component rotations. Positive rotation about the transverse (x) axis corresponds to anterior tilt of the tibial tray. Positive rotation about the longitudinal (y-axis) axis corresponds to internal rotation of the component. Positive rotation about the sagittal axis is equivalent to more subsidence of the lateral than of the medial corner of the tibial component or lateral subsidence and medial lift-off, both resulting in a more pronounced valgus position of the knee.

Radiostereometric examinations were performed in a laboratory designed for RSA examinations (AdoraRSA suite, NRT, Aarhus, Denmark) supplied with Canon CXDI-50RF 160 DPI DR detectors and with use of a uniplanar cage. The postoperative examination was performed after initiation of weightbearing (median 1 day, range 0–8 days), and then at follow-up visits. Tibial tray migration was measured in terms of rotations: anterior (+)/posterior (–) tilt, internal (+)/external (–) rotation, valgus (+)/varus (–) tilt of the knee—not the component) (Figure 1). Translations were measured in terms of medial (+)/lateral (–), proximal (+)/distal (–) and anterior (+)/posterior motions of the center of gravity of the tibial markers in the polyethylene insert. A consistent marker method was used. In addition, we account for maximum lift-off and maximum subsidence of any of 5 standardized points on the tibial tray: 1 medial, 1 lateral, 1 anterior, 1 posteromedial, and 1 posterolateral. The positions of these points on the tibial tray have previously been presented [20]. In the present study the locations of these points were marked on the reference radiograph and their positions on the subsequent radiographic examinations were transformed using the rigid body defined by the polyethylene markers. Maximum lift-off corresponds to the maximum positive translation along the proximal–distal (y) axis of any of the 5 standardized positions on the tray. Correspondingly, maximum subsidence was defined as the lowest value for the y-translation of any of these positions, disregarding its location. The vectorial sum of the translations of any of the 5 standardized points that moved the most represented maximum total point motion (MTPM). The 95% confidence limit of the precision error was calculated and corresponded to 2 standard deviations (SD) of the difference between 55 double examinations and a supposed mean difference of zero. All evaluations were performed with use of the UmRSA software (RSA Biomedical, Umeå, Sweden).

### Missing data and follow up

Follow-up visits were planned for 3, 12, 24, and 60 months after surgery. The analysis was conducted according to a per-protocol approach. Missing patient reported outcome measures (PROM) data were handled according to the validated imputation guidelines for each respective instrument to minimize bias. Patients who missed scheduled follow-up visits were contacted and offered an opportunity to reschedule.

### Statistics

This study planned to recruit 70 knees, anticipating that up to 10 patients might not complete all aspects of the study due to logistical or personal reasons. Power calculations indicate that with 30 knees per group, the study would detect a 4-point difference in OKS with a standard deviation of 5 with 80% power. The magnitude of this difference was at the time point for planning of this study arbitrarily set at this level due to lack of information on what could be regarded as MCID.

The primary analysis compared OKS at 2 years between PSI and CVI with an unadjusted independent samples t-test. A sensitivity analysis using a linear mixed model (LMM) was performed to assess robustness. Secondary outcomes (OKS and other PROMs at additional time points) were analyzed exploratorily using unadjusted independent‑samples t‑tests for continuous variables and Pearson’s chi-square test or Fisher’s exact test for frequencies. Normality was assessed by diagnostic plots. The OKS was analyzed as a change from baseline in a repeated measures linear mixed-effects model (LMM), which included patient identification number as a random effect and time (3, 12, 24, and 60 months), surgical instrument (PSI and CVI), preoperative OKS, preoperative alignment (HKA angle), and interactions between treatment group and by time as fixed-effect factors. An unstructured covariance pattern was utilized. Secondary outcomes, including component stability and migration patterns measured by RSA, were evaluated through a similar model to determine the impact of PSI on fixation of the tibial component (see Keiller et. al 2023 for details) [20]. To keep the direction of migration of non-normalized data we chose not to analyze the logarithm of the absolute values, which should be considered when assessing the results. Estimated means for each group and the overall treatment effect (contrast estimates) with 95% confidence intervals (CI) are presented. The inter-observer reliability of rotational and radiolucent lines measurements was determined by calculating the interclass correlation (ICC) based on a consistency-type two-way random model of respective measurements. IBM SPSS Statistics (IBM Corp, Armonk, NY, USA) was used for statistical analysis.

### Ethics, data sharing plan, funding, use of AI, and disclosures

This study was approved by the Regional Ethics Committee Gothenburg, Sweden (Approval number: 2010-13, 2013-05-27, revised under the Swedish Ethical Review Authority). All participants provided informed consent in accordance with the Helsinki Declaration. No formal data-sharing plan was established at the study’s inception; however, data can be shared upon request, contingent on a data-sharing agreement.

Funding for the research was received from the Swedish State under the agreement between the Swedish government and the county councils, the ALF agreement (721791), Inga Britt and Arne Lundberg’s Research Foundation, the Felix Neubergh Foundation, and partly from Zimmer Biomet, The Netherlands. The funders had no role in the design of the study, data collection, analysis, interpretation of results, or manuscript preparation. Artificial intelligence tools, including ChatGPT (OpenAI, San Francisco, CA, USA) and Microsoft Copilot (Microsoft, Redmond, WA, USA), were used to assist with spelling, grammar, and language clarity. No content, data analysis, or interpretation was generated by these tools The authors declare no other conflicts of interest related to this study. Complete disclosure of interest forms according to ICMJE are available on the article page, doi: 10.2340/17453674.2025.44924

## Results

**Figure 2:**
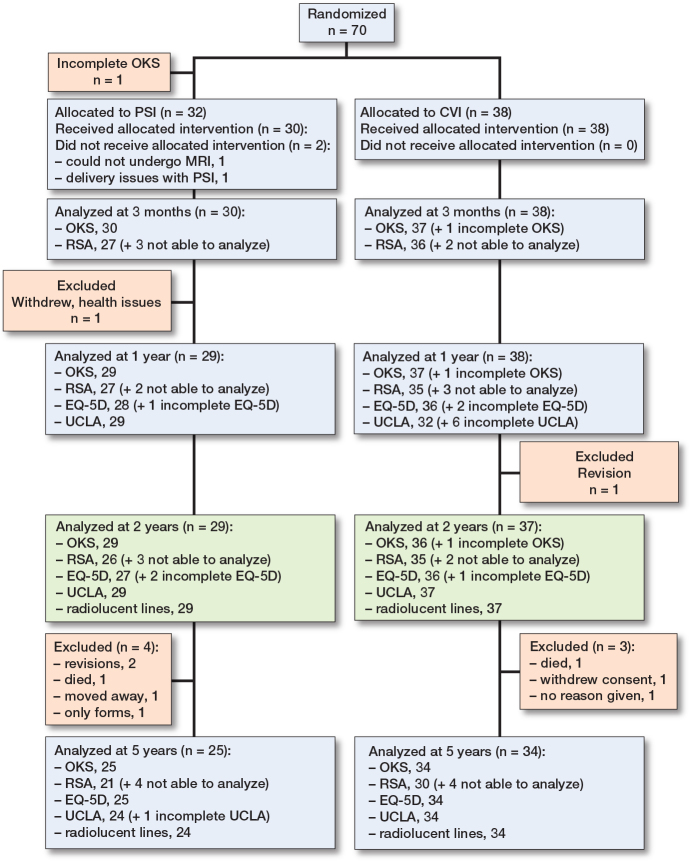
Flow diagram showing patients who underwent follow-up at differing time-points and modalities. Not analyzable were due to hidden or dislodged marker.

66 patients (70 knees) were screened and recruited for the study (Figure 2, Table 1). Due to deviation from the supposed distribution of varus/valgus aligned knees, 32 knees were randomized to PSI and 38 to CVI. Of these, 68 underwent surgery as per protocol, while 2 patients initially allocated to the PSI group did not receive PSI instrumentation. A posterior stabilized implant (LPS) was used in 4 cases (1 PSI and 3 CVI) and a patellar component was used in 1 case (CVI).

**Table 1:** Baseline characteristics. Values are count or mean with standard deviation

Factor	PSI (n = 30)	CVI (n = 38)
Age, years	65 (8)	65 (6)
Weight, kg	86 (13)	83 (12)
Height, cm	170 (8.5)	171 (9.7)
Body mass index	29.7 (3.4)	28.7 (3.4)
Male/female	13/17	16/22
ASA score		
I	7	6
II	17	27
III	3	3
Missing	3	2
Varus/valgus	29/1	33/5
OA grade:		
Ahlbäck 1–2/3–4	18/12	20/18
HKA angle	173.8 (4.7)	173.8 (5.1)
OKS	19.0 (5.8)	19.9 (6.2)
EQ-5D	0.73 (0.09)	0.77 (0.1)
EQ-VAS	44 (29)	58 (20)
UCLA Activity Scale	4.8 (0.8)	4.4 (1.2)
Length of stay, days	3.2 (1.1)	2.9 (1.2)
Bleeding, mL	224 (98)	222 (99)
Surgical time, minutes	104 (17)	102 (20)

ASA = American Society of Anesthesiologists physical status classification system; EQ-5D = EuroQol questionnaire (–0.59 to 1.0); EQ VAS = self-rated overall health on a visual analog scale 0–100; HKA = medial hip-knee-ankle angle; OA = osteoarthritis; OKS = Oxford Knee Score; UCLA = University of California, Los Angeles Activity Scale.

### PROMS and clinical outcomes

**Figure 3:**
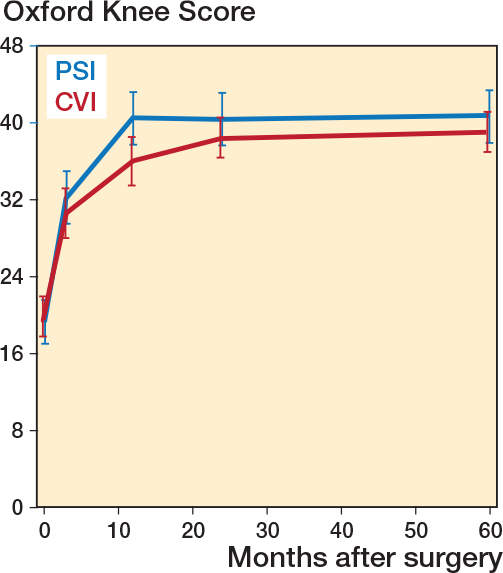
Mean predicted values for Oxford Knee Score with 95% confidence intervals.

Operating time, blood loss, and length of stay were similar in PSI and CVI groups (Table 1). Both groups demonstrated significant improvements in OKS scores at 2 years (OKS mean difference preoperatively vs 2 years: PSI 21.2, CI 17.7–24.6; CVI 18.2, CI 15.7–20.6). No statistically significant difference was observed between the PSI and CVI groups at the 2-year follow-up for the primary outcome measure. Apart from statistically significant differences in favor of PSI in the OKS and EQ-5D at the 1-year visit there were no statistically significant differences between treatment groups in PROMs at other individual visits (Table 2, Figure 3). Repeated measures analysis over the 4 follow-up visits showed no statistically significant difference (contrast estimate) at 2.4 (CI –1.0 to 5.8).

**Table 2:** Differences between groups at up to 5 years. Values for groups are count and mean (SD) or where specified count (%)

Follow up Instrument/test	n	PSI	n	CVI	Mean difference (CI)/OR (CI)
1 year					
Oxford Knee Score ^**b**^	29	41 (8.4)	37	36 (9.1)	4.6 (0.2 to 9.0)
EQ-5D ^**b**^	28	0.90 (0.09)	36	0.85 (0.10)	0.05 (0.001 to 0.10)
EQ-VAS ^**b**^	29	63 (40)	36	70 (22)	–6.8 (–24 to 10)
UCLA Activity Scale ^**b**^	29	4.3 (1.0)	32	4.3 (0.9)	0.3 (–0.5 to 0.5)
2 years					
Oxford Knee Score ^**a**^	29	40.5 (7.4)	36	38.4 (7.1)	2.1 (–1.5 to 5.7)
Improvement from baseline	28	21.2 (8.9)	36	18.2 (7.8)	3.0 (–1.0 to 7.1)
MIC achieved, n (%)	28	25 (89)	36	32 (89)	OR 0.98 (0.40 to 2.4)
Ceiling achieved, n (%) ^**c**^	29	12 (41)	36	11 (31)	OR 0.78 (0.45 to 1.3)
EQ-5D ^**a**^	27	0.90 (0.10)	36	0.88 (0.11)	0.02 (–0.03 to 0.08)
EQ-VAS ^**a**^	29	79 (17)	36	77 (19)	1.4 (–7.4 to 10.3)
UCLA Activity Scale ^**a**^	29	4.2 (1.1)	37	4.1 (1.3)	0.7 (–0.5 to 0.7)
5 years					
Oxford Knee Score ^**a**^	25	40.9 (8.5)	34	39.0 (7.5)	1.9 (–2.3 to 6.1)
EQ-5D ^**a**^	25	0.90 (0.10)	34	0.88 (0.09)	0.02 (–0.03 to 0.07)
EQ-VAS ^**a**^	24	76 (21)	34	75 (15)	0.9 (–8.7 to 10)
UCLA Activity Scale ^**a**^	24	4.3 (1.0)	34	4.4 (1.1)	–0.2 (–0.7 to 0.4)

For abbreviations, see Table 1 and CI = 95% confidence interval; MIC = minimal important change; OR = odds ratio.

a Primary outcome.

b Secondary outcome.

c OKS ceiling is OKS > 43

### Radiography

The tibial components inserted using PSI demonstrated a slightly more pronounced varus position compared with those inserted with CVI. No significant differences were observed in other radiological parameters (Table 3).

**Table 3:** Radiological parameters. Values are count and mean (standard deviation)

Instrument/test	n	PSI	n	CVI		Mean difference (CI)	
HKA, °	30	179 (2.4)	38	179 (2.1)		0.3 (–0.8 to 1.3)	
Component position,°						
Femur, AP view, (varus ≥ 90°)	30	97 (2.3)	38	96 (2.0)	0.8 (–0.3 to 1.8)	
Tibia, AP view, (valgus ≥ 90°)	30	87 (2.2)	38	89 (1.8)	–1.4 (–2.3 to –0.4)	
Femur, lateral view,						
(posterior slope ≥ 90°)	30	89 (4.4)	38	90 (2.5)		–1.6 (–3.3 to 0.1)	
Tibia, lateral view,						
(anterior slope ≥ 90°)	30	83 (2.9)	38	83 (2.6)		0.2 (–1.1 to 1.5)	
Femoral component rotation, + = IR						
epicondylar axis	29	3.8 (2.1)	37	3.6 (2.6)		0.1 (–1.1 to 1.3)	
Tibial component rotation, + = IR						
posterior condyles	29	–6.8 (6.5)	37	–6.3 (6.2)		-0.5 (–3.7 to 2.6)	
lateral landmark ^**a**^	29	87 (8.1)	37	87 (9.0)		–0.2 (–4.5 to 4.0)	
Radiolucent lines, %						
AP view, 2 years	29	11.0 (14.6)	37	7.1 (7.8)		4.2 (–1.8 to 10.3)
Lateral view, 2 years	29	9.9 (14.5)	37	7.1 (10.5)		2.8 (–3.3 to 9.0)
AP view, 5 years	24	9.7 (10.5)	34	7.9 (8.6)		1.9 (–3.2 to 6.9)
Lateral view, 5 years	24	10.2 (17.7)	34	5.9 (10.7)		4.3 (–3.1 to 11.8)

AP = anteroposterior; CI = 95% confidence interval; HKA = medial hip-knee-ankle angle;

IR = internal rotation.

a increasing value = increased IR

### Radiostereometric analysis

The precision of translations varied between 0.08 and 0.20 mm and 0.13 and 0.48 mm for segment and point motion, respectively and the corresponding precision for rotations between 0.11° and 0.28° (Table 4) Both groups exhibited minimal mean micromovements of the tibial components over 5 years, with mean rotations below 0.5° and segment translations ≤ 0.2 mm.

**Table 4:** 95% confidence limits of the precision error based on 55 double examinations

Factor	Precision error CI
Segment translation, mm	
Medial (+)/lateral (–)	0.09
Proximal (+)/distal (–)	0.08
Anterior (+)/posterior (–)	0.20
Point motion, mm	
Maximum lift-off, tibial tray	0.13
Maximum subsidence, tibial tray	0.17
MTPM ^**a**^	0.48
Segment rotations, °	
Anterior (+)/posterior (–) tilt	0.28
Internal (+)/external (–) rotation	0.28
Valgus (+)/varus (–) tilt ^**b**^	0.11

a MTPM (maximum total point motion) = vectorial sum of translations of any of 5 defined points on the tibial tray that moved the most.

b Tilt of the joint.

Tibial components implanted with PSI technique tended to rotate slightly more into varus than those in the control group (Table 5). Analysis of the separate follow up occasions showed no statistically significant difference before the 5 years’ follow-up, at which time point the mean difference between the PSI and CVI groups was –0.28 mm (CI –0.54 to –0.03 mm). In the analysis over time (LMM) the difference (contrast estimate) reached –0.16 (CI –0.30 to –0.03) Table 6).

**Table 5:** Migration (rotations and translations) of tibial component up to 5 years. Values for groups are mean with 95% confidence interval (CI)

Tibial tray	n	PSI (CI)	n	CVI (CI)	Mean difference (CI)
1 year					
Rotation, °					
Anterior (+)/posterior (–) tilt	27	–0.26 (–0.48 to –0.04)	35	–0.19 (–0.36 to –0.02)	–0.08 (–0.34 to 0.20)
Internal (+)/external (–) rotation	27	–0.05 (–0.30 to 0.19)	35	0.01 (–0.13 to 0.14)	–0.06 (–0.32 to 0.20)
Valgus (+)/varus (–) tilt ^**a**^	27	–0.10 (–0.26 to 0.07)	35	–0.02 (–0.10 to 0.06)	–0.08 (–0.25 to 0.09)
Translations, mm					
Medial (+)/lateral (–)	27	0.02 (–0.10 to 0.15)	35	0.01 (–0.05 to 0.07)	0.02 (–0.11 to 0.15)
Proximal (+)/distal (–)	27	–0.04 (–0.09 to 0.01)	35	–0.00 (–0.04 to 0.05)	–0.04 (–0.11 to 0.02)
Anterior (+)/posterior (–)	27	–0.10 (–0.24 to 0.04)	35	–0.09 (–0.16 to –0.02)	–0.01 (–0.16 to 0.13)
MTPM	27	0.70 (0.48 to 0.91)	35	0.54 (0.45 to 0.63)	0.16 (–0.05 to 0.37)
2 years					
Rotations, °					
Anterior (+)/posterior (–) tilt	26	–0.26 (–0.45 to –0.07)	35	–0.20 (–0.38 to –0.01)	–0.06 (–0.33 to 0.20)
Internal (+)/external (–) rotation	26	0.00 (–0.22 to 0.21)	35	0.09 (–0.07 to 0.25)	–0.09 (–0.35 to 0.16)
Valgus (+)/varus (–) tilt ^**a**^	26	–0.11 (–0.27 to 0.05)	35	0.02 (–0.07 to 0.11)	–0.13 (–0.30 to 0.04)
Translations, mm					
Medial (+)/lateral (–)	26	–0.04 (–0.12 to 0.05)	35	–0.05 (–0.11 to 0.01)	0.01 (–0.08 to 0.11)
Proximal (+)/distal (–)	26	–0.07 (–0.15 to 0.01)	35	–0.02 (–0.07 to 0.03)	–0.05 (–0.14 to 0.04)
Anterior (+)/posterior (–)	26	–0.13 (–0.24 to –0.01)	35	–0.08 (–0.15 to 0.00)	–0.05 (–0.18 to 0.08)
MTPM	26	0.65 (0.46 to 0.83)	35	0.62 (0.45 to 0.79)	0.03 (–0.21 to 0.28)
5 years					
Rotations, °					
Anterior (+)/posterior (–) tilt	21	–0.12 (–0.35 to 0.12)	30	–0.41 (–0.66 to –0.15)	0.29 (–0.07 to 0.65)
Internal (+)/external (–) rotation	21	0.24 (–0.02 to 0.50)	30	0.15 (–0.08 to 0.38)	0.09 (–0.25 to 0.43)
Valgus (+)/varus (–) tilt ^**a**^	21	–0.26 (–0.50 to –0.02)	30	0.03 (–0.12 to 0.17)	–0.28 (–0.54 to –0.03)
Translations, mm					
Medial (+)/lateral (–)	21	0.16 (–0.04 to 0.36)	30	0.01 (–0.08 to 0.10)	0.15 (–0.04 to 0.34)
Proximal (+)/distal (–)	21	–0.12 (–0.29 to 0.06)	30	–0.15 (–0.27 to –0.02)	0.03 (–0.17 to 0.23)
Anterior (+)/posterior (–)	21	–0.08 (–0.24 to –0.07)	30	–0.20 (–0.35 to –0.05)	0.12 (–0.08 to 0.33)
MTPM	21	0.88 (0.61 to 1.15)	30	0.71 (0.52 to 0.91)	0.17 (–0.15 to 0.49)

a Tilt of the joint.

MTPM, see Table 4.

**Table 6:** Model estimate of mean least square difference (3 months, 1, 2, and 5 years). Contrast estimate is data for PSI minus data for CVI. Values for groups are estimated mean and 95% confidence interval (CI)

	PSI (CI)	CVI (CI)	Contrast estimate (CI)
Tibial component rotations			
Anterior (+)/posterior (–) tilt	–0.16 (–0.30 to –0.01)	–0.22 (–0.34 to –0.09)	0.55 (–0.13 to 0.25)
Internal (+)/external (–) rotation	–0.01 (–0.16 to 0.15)	0.06 (–0.07 to 0.19)	–0.06 (–0.26 to 0.14)
Valgus (+)/varus (–) tilt ^**a**^	–0.14 (–0.24 to –0.04)	0.02 (–0.07 to 0.11)	–0.16 (–0.30 to –0.03)
Tibial component translations			
Medial (+)/lateral (–)	0.04 (–0.03 to 0.12)	–0.02 (–0.08 to 0.04)	0.06 (–0.03 to 0.16)
Proximal (+)/distal (–)	–0.06 (–0.12 to –0.00)	–0.04 (–0.09 to 0.01)	–0.02 (–0.10 to 0.06)
Anterior (+)/posterior (–)	–0.08 (–0.16 to –0.01)	–0.09 (–0.15 to –0.03)	0.00 (–0.10 to 0.10)
MTPM	0.75 (0.58 to 0.92)	0.57 (0.42 to 0.72)	0.18 (–0.04 to 0.41)
Maximum lift–off			
Proximal (+) translation	0.13 (0.06 to 0.21)	0.10 (0.03 to 0.17)	0.03 (–0.07 to 0.14)
Maximum subsidence			
Distal (–) translation	–0.35 (–0.47 to –0.22)	–0.26 (–0.37 to –0.15)	–0.09 (–0.26 to 0.08)

a Tilt of the joint.

MTPM, see Table 4.

There was no correlation between the magnitude of rotation into varus of the tibial component and the HKA angle (Spearman’s rho: 0.0–0.1) or the position of the tibial component in the coronal plane (Spearman’s rho: –0.1 to 0.2) on any of the follow-up occasions.

**Figure 4:**
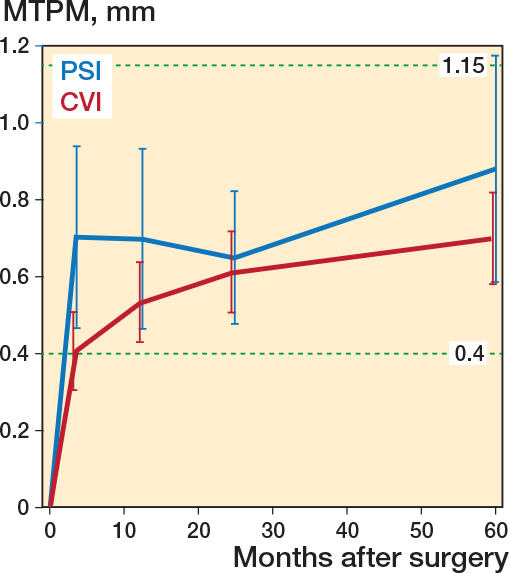
Mean predicted values for maximum total point motion (MTPM). Reference lines at 0.4 and 1.15 represent 1-year thresholds (Puijk et. al 2025).

At 5 years MTPM had reached 0.88 mm in the PSI and 0.71 mm in the CVI group, mainly due to distal migration with a weak tendency to more migration in the PSI group (Figure 4, Table 5). Repeated measures analysis in the LMM showed an estimated mean difference of 0.18 (CI –0.04 to 0.39; Table 6). Analysis of the separate follow-up occasions revealed no statistically significant difference (Tables 5 and 7) except from comparison of data between 1 and 2 years’ follow-up (mean difference 0.13 mm, CI 0.01–0.25; see Supplementary data).

**Table 7:** Migration of defined points on the tibial component up to 5 years. Values for groups are mean, mm (95% confidence interval) or distribution

Tibial tray	n	PSI (CI) or distribution	n	CVI (CI) or distribution	Mean difference (CI)
1 year					
Maximum lift-off					
Location on tibial tray ^**a**^	26	0/13/11/3	35	3/12/15/5	–
Proximal (+) translation	26	0.11 (0.03 to 0.18)	35	0.12 (0.05 to 0.18)	–0.01 (–0.11 to 0.09)
Maximum subsidence					
Location on tibial tray ^**a**^	26	0/13/10/4	35	2/11/13/9	–
Distal (–) translation	26	–0.32 (–0.50 to –0.15)	35	–0.22 (–0.30 to –0.13)	–0.11 (–0.28 to 0.07)
2 years					
Maximum lift-off					
Location on tibial tray ^**a**^	26	0/13/11/2	35	3/15/13/4	–
Proximal (+) translation	26	0.12 (0.03 to 0.21)		0.12 (0.01 to 0.22)	0.01 (–0.14 to 0.15)
Maximum subsidence					
Location on tibial tray ^**a**^	26	0/11/9/6	35	1/12/12/10	–
Distal (–) translation	26	–0.33 (–0.52 to –0.15)	35	–0.24 (–0.32 to –0.15)	–0.10 (–0.28 to 0.08)
5 years					
Maximum lift-off					
Location on tibial tray ^**a**^	21	1/12/7/1	30	3/14/10/3	–
Proximal (+) translation	21	0.14 (0.07 to 0.34)	30	0.06 (–0.07 to 0.18)	0.08 (–0.14 to 0.30)
Maximum subsidence					
Location on tibial tray ^**a**^	21	0/7/11/3	30	0/11/11/8	–
Distal (–) translation	21	–0.51 (–0.76 to –0.25)	30	–0.44 (–0.66 to –0.23)	–0.06 (–0.39 to 0.26)

^a^ Figures indicate number of cases with maximum values observed anteriorly, laterally, medially, postero-laterally or postero-medially. Presence of maximum subsidence or maximum lift-off at the locations of any of the 2 posterior points have been merged.

### Revisions

3 knees were revised during follow-up. 1 knee (CVI) was revised before the 2-year visit due to instability. A further 2 knees (both PSI group) underwent revision prior to the 5-year follow-up. 1 knee was revised to include a patellar component due to patellar tracking issues and swelling and another due to late infection in a 2-stage procedure.

## Discussion

We aimed to compare PSI and CVI in TKA. At 2 years, we found no significant differences in PROMs between PSI and CVI. This finding should be interpreted against the background that the only revision due to instability was performed in the CVI group and before follow-up at 2 years. Evaluation of the overall treatment effect up to 5 years confirmed these results. Based on the primary outcome (OKS at 2 years), no difference was identified between groups, although a clinically important difference is possible based on the limits of the 95% CI exceeding MCID. Nonetheless the OKS was numerically higher in the PSI group throughout the observation period and especially so at 1 year when the difference reached statistical significance, but the mean difference between groups did not exceed the MCID. These findings align with prior studies showing no consistent clinical advantage of PSI in TKA. Like earlier meta-analyses, Hampton et al. (2022) reported minor differences in alignment accuracy with PSI but no corresponding enhancement in functional outcomes or implant survival [5,7,11]. A 10-year follow-up of a multicenter RCT by Theeuwen et al. (2024) and a 5-year follow-up and secondary analysis by Rivrud et. al (2023) reported slight differences in implant positioning but no advantage in patient-reported outcomes or complication rates [6, 12]. The latter study highlighted the limited durability of alignment improvements and reinforced the notion that functional recovery may depend more on other patient or surgical factors than on precision instrumentation.

A statistically significant difference in migration in the coronal tibial alignment was found, with the PSI group showing slightly more varus alignment. However, the difference was small, and group mean values remained within generally accepted thresholds (0–3° varus tilt). Notably, we observed a slightly increased varus tilting of the tibial component up to 5 years, suggesting that increased varus positioning of the tibial tray would result in increased tilting into varus during the postoperative years. There was, however, no correlation between the postoperative varus–valgus position of the tibial component and the subsequent varus–valgus tilting according to RSA during the follow-up period, which rebuts this hypothesis. Our RSA data revealed a slightly higher but statistically insignificant maximum subsidence in the PSI group, somewhat consistent with earlier findings by Ohrn et al. (2018) [9]. However, it contrasts with Teeter et al. (2019) in which the CVI group had a slightly higher mean MTPM at 2 years, although an explicit definition of how MTPM was measured is not provided in the study [4]. The mean values observed in our study were small and below the threshold for unacceptable early migration as defined by Puijk et al. (2025) [21]. The mean rotations around the transverse axis were well below the of 0.8° defined by Gudnason et al. (2017) with 4 tibial components exceeding this value in each group [22]. With regards to MTPM, both the PSI and CVI groups in our study fall within the “at risk” category for future revision according to Puijk et al. Thus, it seems that the risk of future revision due to loosening is about the same in our PSI and CVI groups.

In contrast to some earlier studies [10] we found no differences in coronal alignment (HKA angle). This aligns with findings in a meta-analysis by Mannan et al. (2014), who similarly concluded that PSI does not appear to impact coronal alignment outcomes [8]. While coronal alignment has been linked to implant survival and function [2], research suggests outlier analysis may have minimal impact [23]. Studies that report statistically significant differences in alignment or implant positioning with PSI rarely include PROMs, and when they do, these differences do not correlate with PROM improvements [3,10,12]. Whether this is due to adaptive kinematics, low responsiveness in PROMs, patient variability in the perception of functional improvement, or lack of transferability or validity of implant positioning studies is beyond the scope of this study but remains a valid research topic.

### Strengths

Key strengths of this study were its randomized design, mid-term follow-up, and the comprehensive assessment of secondary outcomes using multiple measurement methods.

### Limitations

The trial allowed for blinding assessors to treatment allocation. However, during follow-up, assessors could access surgical reports to determine the assigned treatment, which introduces the possibility of bias. Additionally, patients were not blinded. The use of new technology such as PSI could introduce a positive bias in favor of PSI and this is a notable concern for PROMS, where scores may be inflated in the PSI group. However, this potential bias did not result in superior clinical outcomes at the 2-year primary endpoint.

Our power calculation was performed over a decade ago, at a time when interpretation of PROM data and the ceiling effect of the OKS in knee arthroplasty outcome evaluation were not as well understood. It was based on previous studies available at that time and did not account for complex variability patterns, which may have underestimated the instrument’s variability. Several studies have since then been published that delineate the MIC, MCID, validity, variability, responsiveness, and ceiling effects of the OKS [14,15,24]. We could not find studies explicitly validating OKS for assessing specific surgical techniques in TKA, as it was originally developed to evaluate overall TKA outcomes [13]. At the time of the study’s inception, it was considered a well-documented PROM with few equivalent alternatives. More recently, the Forgotten Joint Score has gained attention for its lower ceiling effect. In our study, the mean difference in OKS favored the PSI group by 2 points, though this difference was not statistically significant and did not exclude a clinically important difference. The dropout rate was relatively small, but, augmented by an uneven allocation, the sample size became insufficient for detecting a 4-point difference in OKS with 80% power in line with our original power calculation, which needed 30 patients in each group. The difference observed by us is below the later reported MCID and the proportion of patients achieving an improvement of 8 points or more was similar in the 2 groups but may have been affected by the OKS ceiling effect that was present in our study.

The study did not include provisions to exclude any possible learning curve associated with PSI. However, all participating surgeons had extensive experience using conventional instruments to insert the NexGen knee, which could constitute a bias. While investigations into the learning curve associated with PSI in TKA are sparse, 1 study suggests that there is a prolonged learning curve of more than 20 cases [25]. Additionally, our study compared only 1 specific brand of PSI with conventional instrumentation, limiting the generalizability of our findings.

Radiographic analysis presented challenges due to the lack of standardized methods for measuring tibial component rotation [11]. In the absence of a universally accepted standard, we adopted a pragmatic approach to compare rotational alignment between the 2 groups.

### Conclusions

This study did not demonstrate PSI to be superior to CVI in standard TKA based on the primary endpoint at 2 years. Exploratory analysis of secondary outcomes at 5 years revealed no clinically significant differences between the 2 techniques. Although statistically significant differences in early OKS scores, tibial alignment, and migration were observed, these were small, constituted an exploratory analysis, and did not translate into improved mid-term patient outcomes or implant survivorship. The slightly increased varus angulation and tendency for higher MTPM in the PSI group suggest a potential increased risk of loosening, though longer follow-up with larger sample sizes is required to confirm this hypothesis. Given the findings of this study and available long-term data from other studies, PSI does not demonstrate a consistent advantage over CVI in standard TKA.

## Supplementary Material



## Supplementary data

Supplementary Tables 1–11 is available on the articles home page, doi: 10.2340/17453674.2025.44924

## Notes

JHH: data curation, formal analysis, software, validation, visualization, writing – original draft, writing – review & editing. TS: conceptualization, data curation, formal analysis, funding acquisition, investigation, methodology, project administration, resources, supervision, writing – review & editing. BS: data curation, software, writing – review & editing. JK: conceptualization, data curation, formal analysis, funding acquisition, investigation, methodology, project administration, resources, software, supervision, validation, visualization, writing – original draft, writing – review & editing. AN: supervision, writing – review & editing, resources. MM: investigation, supervision, writing – review & editing.

Handling co-editors: Marianne Westberg and Robin Christensen

*Acta* thanks Bart G Pijls and Stephan Maximilian Röhrl for help with peer review of this manuscript.
