# Supplementary material for: Effect of patient-specific instruments compared with conventional instruments in total knee arthroplasty: a randomized controlled trial
Source: Acta Orthop. 2025 Dec 2;96:875–84. doi: 10.2340/17453674.2025.44924 (PMC12673265; doi:10.2340/17453674.2025.44924)
Supplement: Supplementary file 1 [file ActaO-96-44924-s1.pdf]

Table 1. RSA. Migration (rotations and translations) of tibial component up to 3 months with 95% confidence intervals (CI)

	Mean (CI)		Mean difference (CI)
	**PSI**	**CVI**	
**Rotations, degrees**			
Anterior (+)/posterior (–) tilt	–0.06 (–0.30 to 0.18)	–0.07 (–0.16 to –0.02)	0.01 (–0.22 to 0.24)
Internal (+)/external (–) rotation	–0.17 (–0.56 to 0.22)	0.02 (–0.11 to 0.14)	–0.19 (–0.54 to 0.17)
Valgus (+)/varus (–) tilt [a]	–0.08 (–0.21 to 0.07)	0.04 (–0.02 to 0.10)	–0.11 (–0.25 to 0.02)
**Translations, mm**			
Medial (+)/lateral (–) [b]	0.03 (–0.03 to 0.09)	–0.01 (–0.06 to 0.04)	0.04 (–0.03 to 0.12)
Proximal (+)/distal (–) [b]	–0.03 (–0.08 to 0.02)	0.03 (–0.01 to 0.06)	–0.05 (–0.11 to 0.01)
Anterior (+)/posterior (–) [b]	–0.05 (–0.16 to 0.06)	0.01 (–0.04 to 0.06)	–0.06 (–0.17 to 0.05)
MTPM [b, c]	0.70 (0.28 to 1.13)	0.41 (0.33 to 0.48)	0.30 (–0.07 to 0.66)

[a] tilt of the joint.
[b] 3-dimensional translation of the marker in the tibial tray that moved the most.
[c] Vectorial sum of translations along the 3 coordinate axes.

Table 2. RSA. Migration of defined points on the tibial component up to 3 months with 95% confidence intervals (CI)

	**Mean, mm (CI) or distribution [a]**		**Mean difference (CI) (if applicable)**
**Tibial tray**	**PSI**	**CVI**	
**Maximum lift-off**			
Location on tibial tray [a]	2/9/11/5	2/17/13/4	–
Proximal (+) translation	0.16 (0.08 to 0.23)	0.13 (0.09 to 0.18)	0.02 (–0.06 to 0.11)
**Maximum subsidence**			
Location on tibial tray [a]	1/10/12/4	1/12/15/8	–
Distal (–) translation	–0.21 (–0.35 to –0.08)	–0.12 (–0.17 to –0.07)	–0.09 (–0.22 to 0.04)

[a] Numbers indicate number of cases with maximum values observed anteriorly, laterally, medially, postero-laterally or postero-medially
[b] Chi-square test

[c] Mann-Whitney test

Table 3. RSA. Migration (rotations and translations) of tibial component 1 to 2 years with 95% confidence intervals (CI)

	Mean (CI)		Mean difference (CI)
	PSI	CVI	
**Rotations, degrees**			
Anterior (+)/posterior (–) tilt	0.05 (–0.14 to 0.24)	–0.03 (–0.09 to 0.03)	0.08 (–0.09 to 0.25)
Internal (+)/external (–) rotation	0.01 (–0.13 to 0.14)	0.05 (–0.02 to 0.13)	–0.05 (–0.19 to 0.09)
Valgus (+)/varus (–) tilt [a]	–0.01 (–0.04 to 0.02)	0.01 (–0.03 to 0.05)	–0.02 (–0.07 to 0.04)
**Translations, mm**			
Medial (+)/lateral (–) [b]	0.01 (–0.02 to 0.05)	–0.02 (–0.05 to 0.01)	0.03 (–0.02 to 0.07)
Proximal (+)/distal (–) [b]	–0.03 (–0.07 to 0.02)	–0.03 (–0.05 to 0.00)	0.00 (–0.05 to 0.05)
Anterior (+)/posterior (–) [b]	0.00 (–0.11 to 0.12)	–0.01 (–0.04 to 0.03)	0.01 (–0.09 to 0.12)
MTPM [b, c]	0.38 (0.25 to 0.52)	0.25 (0.21 to 0.30)	0.13 (0.01 to 0.25)

[a, b c] See Table1.

Table 4. RSA. Migration of defined points on the tibial component 1 to 2 years with 95% confidence intervals (CI)

	Mean, mm (CI) or distribution [a]		Mean difference (CI) (if applicable)
**Tibial tray**	**PSI**	**CVI**	
**Maximum lift-off**			
Location on tibial tray [a]	0/13/8/6	7/13/9/7	–
Proximal (+) translation	0.01 (–0.02 to 0.04)	0.02 (–0.02 to 0.06)	–0.01–0.06 to 0.05)
**Maximum subsidence**			
Location on tibial tray [a]	1/9/13/4	0/11/11/14	–
Distal (–) translation	–0.09 (–0.14 to –0.049	–0.09 (–0.12 to –0.06)	0.00 (–0.05 to 0.05)

[a] Numbers indicate number of cases with maximum values observed anteriorly, laterally, medially, postero-laterally or postero-medially

Table 5. RSA. Migration (rotations and translations) of tibial component 2 to 5 years with 95% confidence intervals (CI)

	Mean (CI)		Mean difference (CI)
	**PSI**	**CVI**	
**Rotations, degrees**			
Anterior (+)/posterior (–) tilt	–0.03 (–0.22 to 0.16)	–0.02 (–0.15 to 0.12)	–0.01 (–0.23 to 0.21)
Internal (+)/external (–) rotation	0.09 (–0.10 to 0.29)	0.06 (–0.04 to 0.15)	0.04 (–0.16 to 0.23)
Valgus (+)/varus (–) tilt [a]	–0.01 (–0.11 to 0.09)	–0.03 (–0.08 to 0.02)	0.03 (–0.07 to 0.13)
**Translations, mm**			
Medial (+)/lateral (–) [b]	0.02 (–0.03 to 0.06)	0.06 (0.02 to 0.09)	–0.04 (–0.10 to 0.02)
Proximal (+)/distal (–) [b]	–0.07 (–0.14 to 0.01)	–0.03 (–0.14 to 0.01)	–0.04 (–0.12 to 0.04)
Anterior (+)/posterior (–) [b]	–0.04 (–0.13 to 0.05)	–0.01 (–0.06 to 0.04)	–0.03 (–0.13 to 0.06)
MTPM [b, c]	0.46 (0.34 to 0.58)	0.34 (0.27 to 0.41)	0.12 (–0.01 to 0.24)

[a, b c] See Table 1.

Table 6. RSA. Migration of defined points on the tibial component 2 to 5 years with 95% confidence intervals (CI)

	Mean, mm (CI) or distribution [a]		Mean difference (CI) (if applicable)
**Tibial tray**	**PSI**	**CVI**	
**Maximum lift–off**			
Location on tibial tray [a]	3/9/5/4	4/10/14/2	–
Proximal (+) translation	0.05 (–0.01 to 0.10)	0.04 (–0.01 to 0.08)	–0.01 (–0.06 to –0.08)
**Maximum subsidence**			
Location on tibial tray [a]	0/7/8/6	0/12/7/11	–
Distal (–) translation	–0.13 (–0.20 to –0.05)	–0.14 (–0.20 to –0.08)	0.01 (–0.08 to 0.10)

[a] Numbers indicate number of cases with maximum values observed anteriorly, laterally, medially, postero-laterally or postero-medially

Table 7. Model estimate of mean least square difference of RSA parameters at 3 months. Data for PSI minus data for knees operated on with standard instruments (CVI) with 95% confidence intervals (CI)

	**Estimated mean (CI)**		**Contrast estimate (CI)**
	**PSI**	**CVI**	
**Tibial component rotations**			
Anterior (+)/posterior (–) tilt	–0.05 (–0.25 to 0.15)	–0.07 (–0.25 to 0.10)	0.02 (–0.24 to 0.29)
Internal (+)/external (–) rotation	–0.16 (–0.38 to 0.06)	0.01 (–0.18 to 0.20)	–0.17 (–0.46 to 0.12)
Valgus (+)/varus (–) tilt [a]	–0.07 (–0.20 to 0.06)	0.05 (–0.07 to 0.16)	–0.12 (–0.29 to 0.06)
**Tibial component translations**			
Medial (+)/lateral (–) [b]	0.03 (–0.06 to 0.12)	–0.02 (–0.09 to 0.06)	0.05 (–0.07 to 0.17)
Proximal (+)/distal (–) [b]	–0.02 (–0.10 to 0.05)	0.02 (–0.05 to 0.09)	–0.04 (–0.14 to 0.06)
Anterior (+)/posterior (–) [b]	–0.05 (–0.16 to 0.05)	0.01 (–0.08 to 0.11)	–0.06 (–0.20 to 0.08)
MTPM [b, c]	0.71 (0.50 to 0.92)	0.40 (0.22 to 0.59)	0.30 (0.02 to 0.59)
**Maximum lift-off**			
Proximal (+) translation	0.17 (0.07 to 0.26)	0.13 (0.04 to 0.21)	0.04 (–0.09 to 0.17)
**Maximum subsidence**			
Distal (–) translation	–0.22 (–0.36 to –0.07)	–0.12 (–0.25 to –0.03)	–0.09 (–0.28 to 0.10)

[a, b, c] See Table 1.

Table 8. Model estimate of mean least square difference of RSA parameters at 1 year. Data for PSI minus data for knees operated with standard instruments (CVI) with 95% confidence intervals (CI)

	Estimated mean (CI)		Contrast estimate (CI)
	PSI	CVI	
**Tibial component rotations**			
Anterior (+)/posterior (–) tilt	–0.23 (–0.44 to –0.03)	–0.19 (–0.36 to –0.01)	–0.05 (–0.32 to 0.22)
Internal (+)/external (–) rotation	–0.05 (–0.28 to 0.17)	0.00 (–0.19 to 0.19)	–0.05 (–0.35 to 0.24)
Valgus (+)/varus (–) tilt [a]	–0.10 (–0.24 to 0.03)	–0.01 (–0.14 to 0.10)	–0.08 (–0.26 to 0.10)
**Tibial component translations**			
Medial (+)/lateral (–) [b]	0.03 (–0.06 to 0.12)	0.00 (–0.08 to 0.08)	0.02 (–0.10 to 0.14)
Proximal (+)/distal (–) [b]	–0.04 (–0.12 to 0.04)	0.00 (–0.07 to 0.07)	–0.04 (–0.14 to 0.07)
Anterior (+)/posterior (–) [b]	–0.10 (–0.20 to 0.01)	–0.09 (–0.18 to –0.00)	–0.01 (–0.15 to 0.14)
MTPM [b, c]	0.71 (0.50 to 0.92)	0.54 (0.36 to 0.73)	0.16 (–0.12 to 0.45)
**Maximum lift-off**			
Proximal (+) translation	0.11 (0.01 to 0.20)	0.11 (–0.03 to 0.19)	0.00 (–0.13 to 0.13)
**Maximum subsidence**			
Distal (–) translation	–0.32 (–0.47 to –0.18)	–0.22 (–0.34 to –0.09)	–0.11 (–0.30 to 0.09)

[a, b c] See Table 1.

Table 9. Model estimate of mean least square difference of RSA parameters at 2 years. Data for PSI minus data for knees operated on with standard instruments (CVI) with 95% confidence intervals (CI)

	Estimated mean (CI)		Contrast estimate (CI)
	PSI	CVI	
**Tibial component rotations**			
Anterior (+)/posterior (–) tilt	–0.26 (–0.46 to –0.05)	–0.20 (–0.38 to –0.03)	–0.06 (–0.32 to 0.22)
Internal (+)/external (–) rotation	–0.02 (–0.25 to 0.20)	0.09 (–0.11 to 0.28)	–0.11 (–0.41 to 0.19)
Valgus (+)/varus (–) tilt [a]	–0.15 (–0.28 to –0.13)	0.02 (–0.06 to 0.10)	–0.17 (–0.34 to 0.13)
**Tibial component translations**			
Medial (+)/lateral (–) [b]	–0.11 (–0.10 to 0.08)	–0.06 (–0.13 to 0.03)	0.04 (–0.08 to 0.17)
Proximal (+)/distal (–) [b]	–0.06 (–0.14 to 0.01)	–0.03 (–0.10 to 0.04)	–0.03 (–0.14 to 0.07)
Anterior (+)/posterior (–) [b]	–0.12 (–0.23 to –0.01)	–0.08 (–0.17 to –0.02)	–0.05 (–0.19 to 0.10)
MTPM [b, c]	0.72 (0.50 to 0.93)	0.62 (0.43 to 0.81)	0.10 (–0.19 to 0.38)
**Maximum lift-off**			
Proximal (+) translation	0.14 (0.04 to 0.23)	0.10 (0.02 to 0.23)	0.03 (–0.10 to 0.16)
**Maximum subsidence**			
Distal (–) translation	–0.36 (–0.50 to –0.21)	–0.24 (–0.37 to –0.12)	–0.11 (–0.31 to 0.08)

[a, b c] See Table 1.

Table 10. Model estimate of mean least square difference of RSA parameters at 5 years. Data for PSI minus data for knees operated on with standard instruments (CVI) with 95% confidence intervals (CI)

	Estimated mean (CI)		Contrast estimate (CI)
	PSI	CVI	
**Tibial component rotations**			
Anterior (+)/posterior (–) tilt	–0.20 (–0.38 to –0.03)	–0.41 (–0.60 to –0.22)	0.31 (0.02 to 0.61)
Internal (+)/external (–) rotation	0.22 (–0.03 to 0.47)	0.14 (–0.07 to 0.35)	0.08 (–0.24 to 0.40)
Valgus (+)/varus (–) tilt [a]	–0.23 (–0.38 to –0.09)	0.04 (–0.08 to 0.16)	–0.27 (–0.46 to –0.08)
**Tibial component translations**			
Medial (+)/lateral (–) [b]	0.13 (0.03 to 0.23)	–0.01 (–0.09 to 0.07)	0.14 (0.01 to 0.27)
Proximal (+)/distal (–) [b]	–0.12 (–0.20 to –0.03)	–0.15 (–0.22 to 0.08)	0.03 (–0.08 to 0.14)
Anterior (+)/posterior (–) [b]	–0.08 (–0.20 to –0.04)	–0.20 (–0.30 to –0.10)	0.12 (–0.03 to 0.28)
MTPM [b, c]	0.88 (0.65 to 1.11)	0.71 (0.52 to 0.91)	0.16 (–0.14 to 0.461)
**Maximum lift-off**			
Proximal (+) translation	0.13 (0.04 to 0.23)	0.06 (–0.03 to 0.15)	0.07 (–0.07 to 0.21)
**Maximum subsidence**			
Distal (–) translation	–0.49 (–0.65 to –0.34)	–0.44 (–0.58 to –0.31)	–0.05 (–0.26 to 0.15)

[a, b c] See Table 1.

Table 11. Loss to follow up (LTF) demographics

	Baseline		LTF 2 year				LTF 5 years			
	PSI (n=30)	***CVI (n=38)***	PSI		CVI		PSI (n=5)		CVI (n=4)	
**Factor**	Mean (SD)/N	Mean/N	Mean/N	SD/N	Mean/N	SD/N	Mean/N	SD/N	Mean/N	SD/N
**Age, years**	65 (8)	65 (6)	59		63		62	5	70	5
**Weight, kg**	86 (13)	83 (12)	95		62		90	15	80	17
**Height, cm**	170 (8.5)	171 (9.7)	178		164		173	4	173	12
**BMI**	29.7 (3.4)	28.7 (3.4)	30		23,1		30	4,5	26,3	2,5
**Male/female**	13/17	16/22		0/1		0/1		3/2		2/2
**ASA**										
**I**	7	6						1		1
**II**	17	27						3		2
**III**	3	3						0		1
**missing**	3	2		1		2		1		0
**Varus/valgus**	29/1	33/5		1/0		1/0		5/0		3/1
**OA Grade. Ahlbäck 1-2/3-4**	18/12	20/18		1/0		1/0		4/1		3/1
**HKA angle**	173.8 (4.7)	173.8 (5.1)	172		168		174	5	170,1	5
**Preop OKS/Last OKS (LTF)**	19.0 (5.8)	19.9 (6.2)	15/40 (3mo)		19/9 (1yr)		19,8/32	6,5/8,6	20,5/35,3	5,2/13,6
Length of stay, days	*3.2 (1.1)*	*2.9 (1.2)*	5		2		3,6	1,3	1,75	0,5
Bleeding, mL	*224 (98)*	*222 (99)*	300		350		280	148	263	75
Surgical time, minutes	*104 (17)*	*102 (20)*	113		118		112	6	102	19
